# (*E*)-*N*′-[(*E*)-2-Methyl­pent-2-enyl­idene]isonicotinohydrazide

**DOI:** 10.1107/S1600536810019434

**Published:** 2010-05-29

**Authors:** H. S. Naveenkumar, Amirin Sadikun, Pazilah Ibrahim, Wan-Sin Loh, Hoong-Kun Fun

**Affiliations:** a School of Pharmaceutical Sciences, Universiti Sains Malaysia, 11800 USM, Penang, Malaysia; b X-ray Crystallography Unit, School of Physics, Universiti Sains Malaysia, 11800 USM, Penang, Malaysia

## Abstract

The asymmetric unit of the title Schiff base compound, C_12_H_15_N_3_O, contains two crystallographically independent mol­ecules, with both existing in an *E* configuration with respect to the C=N double bonds. In the crystal structure, inter­molecular N—H⋯N and C—H⋯O hydrogen bonds link the mol­ecules into a three-dimensional network.

## Related literature

For the applications of isoniazid derivatives, see: Janin (2007 ▶); Maccari *et al.* (2005 ▶); Slayden & Barry (2000 ▶). For the bio­logical activity of Schiff bases, see: Kahwa *et al.* (1986 ▶). For related structures, see: Naveenkumar *et al.* (2009 ▶); Naveenkumar, Sadikun, Ibrahim, Quah & Fun (2010 ▶); Naveenkumar, Sadikun, Ibrahim, Yeap & Fun (2010 ▶); Shi (2005 ▶). For bond-length data, see: Allen *et al.* (1987 ▶). For the stability of the temperature controller used for the data collection, see: Cosier & Glazer (1986 ▶). For the synthesis, see: Lourenco *et al.* (2008 ▶).
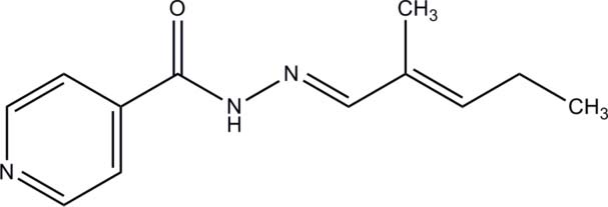


## Experimental

### 

#### Crystal data

C_12_H_15_N_3_O
*M* *_r_* = 217.27
Monoclinic, 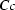

*a* = 19.809 (4) Å
*b* = 8.3459 (15) Å
*c* = 16.021 (3) Å
β = 119.825 (3)°
*V* = 2297.7 (7) Å^3^
*Z* = 8
Mo *K*α radiation
μ = 0.08 mm^−1^
*T* = 100 K
0.54 × 0.20 × 0.10 mm

#### Data collection

Bruker APEXII DUO CCD area-detector diffractometer
Absorption correction: multi-scan (*SADABS*; Bruker, 2009 ▶) *T*_min_ = 0.957, *T*_max_ = 0.992
12644 measured reflections
3396 independent reflections
2883 reflections with *I* > 2σ(*I*)
*R*_int_ = 0.044

#### Refinement

*R*[*F* ^2^ > 2σ(*F* ^2^)] = 0.073
*wR*(*F* ^2^) = 0.225
*S* = 1.03
3396 reflections
301 parameters
2 restraints
H atoms treated by a mixture of independent and constrained refinement
Δρ_max_ = 1.15 e Å^−3^
Δρ_min_ = −0.47 e Å^−3^

### 

Data collection: *APEX2* (Bruker, 2009 ▶); cell refinement: *SAINT* (Bruker, 2009 ▶); data reduction: *SAINT*; program(s) used to solve structure: *SHELXTL* (Sheldrick, 2008 ▶); program(s) used to refine structure: *SHELXTL*; molecular graphics: *SHELXTL*; software used to prepare material for publication: *SHELXTL* and *PLATON* (Spek, 2009 ▶).

## Supplementary Material

Crystal structure: contains datablocks global, I. DOI: 10.1107/S1600536810019434/is2553sup1.cif

Structure factors: contains datablocks I. DOI: 10.1107/S1600536810019434/is2553Isup2.hkl

Additional supplementary materials:  crystallographic information; 3D view; checkCIF report

## Figures and Tables

**Table 1:** Hydrogen-bond geometry (Å, °)

*D*—H⋯*A*	*D*—H	H⋯*A*	*D*⋯*A*	*D*—H⋯*A*
N2*B*—H1*NB*⋯N1*A*^i^	0.95 (6)	2.08 (6)	2.973 (5)	156 (5)
N2*A*—H1*NA*⋯N1*B*^ii^	0.83 (6)	2.26 (6)	3.005 (5)	148 (5)
C7*B*—H7*BA*⋯O1*A*^iii^	0.93	2.51	3.171 (5)	129
C12*A*—H12*B*⋯O1*B*	0.96	2.48	3.433 (5)	173

Symmetry codes: (i) 
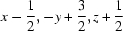
; (ii) 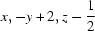; (iii) 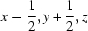.

**Fig. 1.**
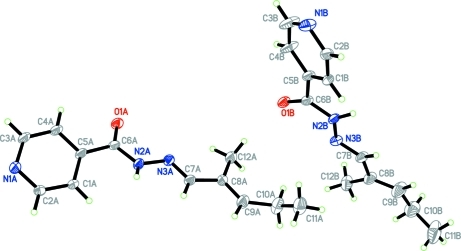
The molecular structure of the title compound, showing 50% probability displacement ellipsoids and the atom-numbering scheme.

**Fig. 2.**
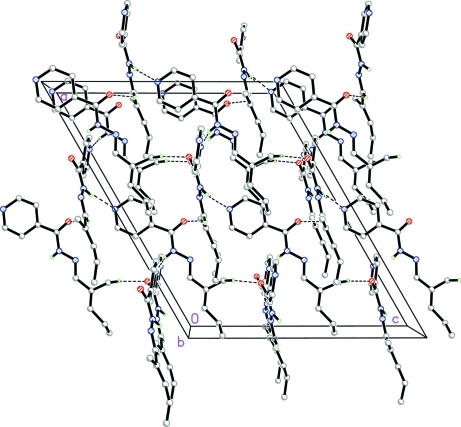
The crystal packing of the title compound, viewed along the b axis. Intermolecular interactions are shown as dashed lines. H atoms not involved in the intermolecular interactions (dashed lines) have been omitted for clarity.

## supplementary crystallographic information

### Comment

In the search of new compounds, isoniazid derivatives have been found to possess potential tuberculostatic activity (Janin, 2007; Maccari *et al.*, 2005; Slayden *et al.*, 2000). Schiff bases have attracted much attention because of their biological activity (Kahwa *et al.*, 1986). As a part of a current work of synthesis of (*E*)-*N*'-substituted isonicotinohydrazide derivatives, in this paper we present the crystal structure of the title compound.

The title Schiff base compound (Fig. 1), consists of two crystallographically independent molecules (molecule A & B). In the molecules, both pyridine rings (C1A/C2A/N1A/C3A–C5A & C1B/C2B/N1B/C3B–C5B) are approximately planar with maximum deviations of 0.006 (5) Å at atom C4A and 0.007 (6) Å at atom C3B. The molecules exist in an *E* configuration with respect to the C7A═N3A and C7B═N3B double bonds. Bond lengths (Allen *et al.*, 1987) and the angles of the title compound are within the normal range and are closely related to comparable structures (Naveenkumar *et al.*, 2009; Naveenkumar, Sadikun, Ibrahim, Quah & Fun, 2010; Naveenkumar, Sadikun, Ibrahim, Yeap & Fun, 2010; Shi, 2005).

In the crystal packing (Fig. 2), intermolecular N2B—H1NB···N1A, N2A—H1NA···N1B, C7B—H7BA···O1A and C12A—H12B···O1B hydrogen bonds (Table 1) link the molecules into a three-dimensional network.

### Experimental

The isoniazid derivative was prepared following the procedure by Lourenco *et al.* (2008). The titled compound was prepared by reaction between the 2-methyl-2-pentenal (1.0 eq) with isoniazid (1.0 eq) in ethanol/water. After stirring for 1-3 h at room temperature, the resulting mixture was concentrated under reduced pressure. The residue, purified by washing with cold ethanol and ethyl ether, afforded the pure derivative. The colourless single crystal suitable for X-ray analysis was obtained by recrystalization with methanol.

### Refinement

H1NA and H1NB were located from a difference Fourier map and were refined freely [N–H = 0.83 (6) or 0.94 (6) Å]. The remaining H atoms were positioned geometrically (C–H = 0.93–0.97 Å) and were refined using a riding model, with *U*_iso_(H) = 1.2 or 1.5 *U*_eq_(C). A rotating group model was applied to all the methyl groups. In the final difference Fourier map, the highest peak is 1.08 Å from atom H11E and the deepest hole is 0.59 Å from atom C9B. In the absence of significant anomalous dispersion, 2998 Friedel pairs were merged before the final refinement.

### Crystal data

**Table:**

C_12_H_15_N_3_O	*F*(000) = 928
*M**_r_* = 217.27	*D*_x_ = 1.256 Mg m^−^^3^
Monoclinic, *C**c*	Mo *K*α radiation, λ = 0.71073 Å
Hall symbol: C -2yc	Cell parameters from 3734 reflections
*a* = 19.809 (4) Å	θ = 2.7–30.1°
*b* = 8.3459 (15) Å	µ = 0.08 mm^−^^1^
*c* = 16.021 (3) Å	*T* = 100 K
β = 119.825 (3)°	Plate, colourless
*V* = 2297.7 (7) Å^3^	0.54 × 0.20 × 0.10 mm
*Z* = 8	

### Data collection

**Table:**

Bruker APEXII DUO CCD area-detector diffractometer	3396 independent reflections
Radiation source: fine-focus sealed tube	2883 reflections with *I* > 2σ(*I*)
graphite	*R*_int_ = 0.044
φ and ω scans	θ_max_ = 30.2°, θ_min_ = 2.4°
Absorption correction: multi-scan (*SADABS*; Bruker, 2009)	*h* = −28→27
*T*_min_ = 0.957, *T*_max_ = 0.992	*k* = −11→11
12644 measured reflections	*l* = −22→22

### Refinement

**Table:**

Refinement on *F*^2^	Primary atom site location: structure-invariant direct methods
Least-squares matrix: full	Secondary atom site location: difference Fourier map
*R*[*F*^2^ > 2σ(*F*^2^)] = 0.073	Hydrogen site location: inferred from neighbouring sites
*wR*(*F*^2^) = 0.225	H atoms treated by a mixture of independent and constrained refinement
*S* = 1.03	*w* = 1/[σ^2^(*F*_o_^2^) + (0.158*P*)^2^ + 2.1081*P*] where *P* = (*F*_o_^2^ + 2*F*_c_^2^)/3
3396 reflections	(Δ/σ)_max_ = 0.001
301 parameters	Δρ_max_ = 1.15 e Å^−^^3^
2 restraints	Δρ_min_ = −0.47 e Å^−^^3^

### Special details

**Table:**

Experimental. The crystal was placed in the cold stream of an Oxford Cryosystems Cobra open-flow nitrogen cryostat (Cosier & Glazer, 1986) operating at 100.0 (1) K.
Geometry. All esds (except the esd in the dihedral angle between two l.s. planes) are estimated using the full covariance matrix. The cell esds are taken into account individually in the estimation of esds in distances, angles and torsion angles; correlations between esds in cell parameters are only used when they are defined by crystal symmetry. An approximate (isotropic) treatment of cell esds is used for estimating esds involving l.s. planes.
Refinement. Refinement of *F*^2^ against ALL reflections. The weighted *R*-factor wR and goodness of fit *S* are based on *F*^2^, conventional *R*-factors *R* are based on *F*, with *F* set to zero for negative *F*^2^. The threshold expression of *F*^2^ > σ(*F*^2^) is used only for calculating *R*-factors(gt) etc. and is not relevant to the choice of reflections for refinement. *R*-factors based on *F*^2^ are statistically about twice as large as those based on *F*, and *R*- factors based on ALL data will be even larger.

### Fractional atomic coordinates and isotropic or equivalent isotropic displacement parameters (Å^2^)

**Table:**

	*x*	*y*	*z*	*U*_iso_*/*U*_eq_	
O1A	0.45226 (16)	0.4174 (4)	0.2514 (2)	0.0279 (6)	
N1A	0.50282 (19)	0.5795 (5)	−0.0143 (2)	0.0270 (7)	
N2A	0.33160 (18)	0.4903 (4)	0.1323 (2)	0.0222 (6)	
N3A	0.29513 (18)	0.4476 (4)	0.1835 (2)	0.0222 (6)	
C1A	0.3923 (2)	0.5171 (5)	0.0042 (2)	0.0228 (7)	
H1AA	0.3391	0.4982	−0.0247	0.027*	
C2A	0.4267 (2)	0.5523 (5)	−0.0517 (3)	0.0245 (7)	
H2AA	0.3950	0.5570	−0.1182	0.029*	
C3A	0.5470 (2)	0.5727 (6)	0.0815 (3)	0.0332 (9)	
H3AA	0.6000	0.5926	0.1087	0.040*	
C4A	0.5184 (2)	0.5376 (6)	0.1427 (3)	0.0307 (8)	
H4AA	0.5516	0.5322	0.2089	0.037*	
C5A	0.4391 (2)	0.5108 (5)	0.1032 (3)	0.0233 (7)	
C6A	0.4089 (2)	0.4684 (5)	0.1703 (2)	0.0219 (7)	
C7A	0.2215 (2)	0.4758 (4)	0.1376 (3)	0.0224 (6)	
H7AA	0.1994	0.5197	0.0762	0.027*	
C8A	0.1715 (2)	0.4418 (5)	0.1777 (3)	0.0256 (7)	
C9A	0.0963 (3)	0.4814 (6)	0.1240 (3)	0.0356 (9)	
H9AA	0.0812	0.5247	0.0638	0.043*	
C10A	0.0337 (3)	0.4641 (7)	0.1499 (4)	0.0429 (11)	
H10A	0.0568	0.4299	0.2164	0.052*	
H10B	−0.0026	0.3818	0.1099	0.052*	
C11A	−0.0096 (3)	0.6178 (7)	0.1369 (4)	0.0448 (12)	
H11A	−0.0508	0.6005	0.1510	0.067*	
H11B	−0.0311	0.6539	0.0717	0.067*	
H11C	0.0254	0.6975	0.1798	0.067*	
C12A	0.2060 (2)	0.3646 (6)	0.2752 (3)	0.0301 (8)	
H12A	0.1734	0.2774	0.2727	0.045*	
H12B	0.2096	0.4422	0.3214	0.045*	
H12C	0.2570	0.3248	0.2936	0.045*	
O1B	0.20324 (15)	0.6331 (3)	0.4358 (2)	0.0268 (6)	
N1B	0.2523 (2)	1.2198 (4)	0.5118 (3)	0.0330 (8)	
N2B	0.08523 (17)	0.7236 (4)	0.4095 (2)	0.0236 (6)	
N3B	0.04966 (18)	0.5750 (4)	0.3851 (2)	0.0241 (6)	
C1B	0.14199 (19)	1.0456 (5)	0.4260 (2)	0.0219 (7)	
H1BA	0.0886	1.0349	0.3852	0.026*	
C2B	0.1756 (2)	1.1958 (5)	0.4529 (3)	0.0256 (7)	
H2BA	0.1434	1.2851	0.4291	0.031*	
C3B	0.2969 (3)	1.0896 (6)	0.5452 (4)	0.0464 (13)	
H3BA	0.3499	1.1039	0.5869	0.056*	
C4B	0.2692 (2)	0.9350 (5)	0.5217 (4)	0.0392 (11)	
H4BA	0.3030	0.8483	0.5459	0.047*	
C5B	0.1895 (2)	0.9105 (4)	0.4610 (3)	0.0241 (7)	
C6B	0.1606 (2)	0.7417 (4)	0.4338 (2)	0.0230 (7)	
C7B	−0.0233 (2)	0.5835 (5)	0.3572 (3)	0.0286 (8)	
H7BA	−0.0463	0.6834	0.3502	0.034*	
C8B	−0.0706 (2)	0.4398 (5)	0.3366 (4)	0.0354 (10)	
C9B	−0.1419 (3)	0.4525 (7)	0.3267 (5)	0.0529 (15)	
H9BA	−0.1580	0.5559	0.3297	0.063*	
C10B	−0.1989 (3)	0.3214 (7)	0.3113 (5)	0.0497 (13)	
H10C	−0.1876	0.2290	0.2836	0.060*	
H10D	−0.1926	0.2896	0.3730	0.060*	
C11B	−0.2777 (4)	0.3700 (8)	0.2492 (8)	0.072 (2)	
H11D	−0.3123	0.2879	0.2476	0.108*	
H11E	−0.2861	0.3870	0.1855	0.108*	
H11F	−0.2876	0.4677	0.2729	0.108*	
C12B	−0.0352 (3)	0.2825 (5)	0.3361 (4)	0.0387 (10)	
H12D	−0.0744	0.2008	0.3132	0.058*	
H12E	0.0050	0.2568	0.4002	0.058*	
H12F	−0.0132	0.2886	0.2946	0.058*	
H1NB	0.059 (3)	0.808 (7)	0.421 (4)	0.023 (11)*	
H1NA	0.306 (3)	0.543 (7)	0.082 (4)	0.028 (13)*	

### Atomic displacement parameters (Å^2^)

**Table:**

	*U*^11^	*U*^22^	*U*^33^	*U*^12^	*U*^13^	*U*^23^
O1A	0.0188 (12)	0.0390 (16)	0.0207 (12)	0.0013 (11)	0.0059 (10)	0.0045 (11)
N1A	0.0189 (14)	0.0374 (18)	0.0235 (15)	−0.0008 (12)	0.0098 (12)	0.0006 (13)
N2A	0.0157 (13)	0.0295 (15)	0.0201 (14)	0.0012 (11)	0.0079 (11)	0.0049 (12)
N3A	0.0198 (14)	0.0243 (14)	0.0219 (13)	−0.0011 (11)	0.0099 (11)	0.0020 (11)
C1A	0.0157 (14)	0.0303 (17)	0.0187 (15)	−0.0021 (12)	0.0056 (12)	−0.0013 (13)
C2A	0.0175 (15)	0.0339 (19)	0.0173 (14)	0.0015 (13)	0.0051 (12)	0.0014 (13)
C3A	0.0150 (15)	0.055 (3)	0.0224 (17)	−0.0095 (16)	0.0041 (13)	0.0000 (17)
C4A	0.0162 (16)	0.048 (2)	0.0202 (16)	−0.0066 (15)	0.0030 (13)	0.0004 (15)
C5A	0.0122 (14)	0.0301 (17)	0.0211 (15)	−0.0009 (12)	0.0034 (12)	0.0021 (13)
C6A	0.0161 (14)	0.0251 (16)	0.0195 (15)	−0.0022 (12)	0.0051 (12)	−0.0028 (12)
C7A	0.0184 (15)	0.0255 (16)	0.0196 (14)	0.0002 (12)	0.0067 (12)	0.0020 (13)
C8A	0.0186 (16)	0.0363 (19)	0.0204 (16)	0.0034 (14)	0.0087 (13)	0.0004 (14)
C9A	0.0235 (19)	0.052 (3)	0.0292 (19)	0.0060 (18)	0.0117 (16)	0.0065 (19)
C10A	0.030 (2)	0.050 (3)	0.052 (3)	0.006 (2)	0.024 (2)	0.008 (2)
C11A	0.038 (3)	0.050 (3)	0.052 (3)	0.009 (2)	0.026 (2)	0.005 (2)
C12A	0.0236 (17)	0.040 (2)	0.0255 (17)	0.0012 (15)	0.0113 (14)	0.0032 (16)
O1B	0.0165 (11)	0.0251 (12)	0.0306 (13)	0.0023 (9)	0.0056 (10)	−0.0032 (11)
N1B	0.0257 (16)	0.0244 (15)	0.0346 (17)	−0.0034 (13)	0.0042 (14)	−0.0038 (14)
N2B	0.0130 (12)	0.0264 (15)	0.0249 (14)	−0.0001 (11)	0.0046 (11)	−0.0037 (12)
N3B	0.0202 (14)	0.0238 (14)	0.0230 (14)	−0.0024 (11)	0.0066 (12)	−0.0003 (11)
C1B	0.0153 (14)	0.0267 (17)	0.0189 (14)	0.0028 (12)	0.0049 (12)	0.0007 (12)
C2B	0.0231 (16)	0.0265 (17)	0.0236 (15)	0.0000 (13)	0.0088 (13)	−0.0017 (13)
C3B	0.0202 (18)	0.031 (2)	0.055 (3)	−0.0013 (16)	−0.0055 (18)	−0.008 (2)
C4B	0.0148 (16)	0.0258 (18)	0.050 (3)	0.0010 (13)	−0.0046 (16)	−0.0063 (17)
C5B	0.0164 (14)	0.0235 (16)	0.0236 (16)	0.0006 (12)	0.0034 (13)	−0.0033 (13)
C6B	0.0181 (15)	0.0240 (16)	0.0200 (14)	−0.0001 (12)	0.0043 (12)	−0.0018 (12)
C7B	0.0191 (15)	0.0243 (17)	0.0342 (19)	−0.0018 (13)	0.0071 (14)	−0.0075 (15)
C8B	0.0235 (18)	0.0281 (19)	0.050 (3)	−0.0089 (15)	0.0144 (18)	−0.0160 (18)
C9B	0.032 (2)	0.040 (3)	0.081 (4)	−0.010 (2)	0.023 (3)	−0.021 (3)
C10B	0.041 (3)	0.041 (3)	0.064 (3)	−0.006 (2)	0.023 (3)	−0.006 (2)
C11B	0.055 (4)	0.044 (3)	0.137 (8)	−0.010 (3)	0.063 (5)	−0.020 (4)
C12B	0.036 (2)	0.0262 (19)	0.063 (3)	−0.0065 (16)	0.032 (2)	−0.0093 (19)

### Geometric parameters (Å, °)

**Table:**

O1A—C6A	1.223 (5)	O1B—C6B	1.228 (5)
N1A—C2A	1.337 (5)	N1B—C3B	1.333 (6)
N1A—C3A	1.338 (5)	N1B—C2B	1.346 (5)
N2A—C6A	1.350 (4)	N2B—C6B	1.350 (4)
N2A—N3A	1.382 (4)	N2B—N3B	1.383 (4)
N2A—H1NA	0.83 (6)	N2B—H1NB	0.94 (6)
N3A—C7A	1.287 (5)	N3B—C7B	1.284 (5)
C1A—C5A	1.384 (5)	C1B—C2B	1.384 (5)
C1A—C2A	1.399 (5)	C1B—C5B	1.395 (5)
C1A—H1AA	0.9300	C1B—H1BA	0.9300
C2A—H2AA	0.9300	C2B—H2BA	0.9300
C3A—C4A	1.385 (6)	C3B—C4B	1.379 (6)
C3A—H3AA	0.9300	C3B—H3BA	0.9300
C4A—C5A	1.389 (5)	C4B—C5B	1.397 (5)
C4A—H4AA	0.9300	C4B—H4BA	0.9300
C5A—C6A	1.510 (5)	C5B—C6B	1.502 (5)
C7A—C8A	1.451 (5)	C7B—C8B	1.456 (6)
C7A—H7AA	0.9300	C7B—H7BA	0.9300
C8A—C9A	1.340 (5)	C8B—C9B	1.344 (7)
C8A—C12A	1.503 (5)	C8B—C12B	1.490 (6)
C9A—C10A	1.496 (6)	C9B—C10B	1.502 (8)
C9A—H9AA	0.9300	C9B—H9BA	0.9300
C10A—C11A	1.499 (8)	C10B—C11B	1.431 (10)
C10A—H10A	0.9700	C10B—H10C	0.9700
C10A—H10B	0.9700	C10B—H10D	0.9700
C11A—H11A	0.9600	C11B—H11D	0.9600
C11A—H11B	0.9600	C11B—H11E	0.9600
C11A—H11C	0.9600	C11B—H11F	0.9600
C12A—H12A	0.9600	C12B—H12D	0.9600
C12A—H12B	0.9600	C12B—H12E	0.9600
C12A—H12C	0.9600	C12B—H12F	0.9600
			
C2A—N1A—C3A	117.0 (3)	C3B—N1B—C2B	116.8 (4)
C6A—N2A—N3A	120.5 (3)	C6B—N2B—N3B	121.1 (3)
C6A—N2A—H1NA	121 (4)	C6B—N2B—H1NB	119 (3)
N3A—N2A—H1NA	117 (4)	N3B—N2B—H1NB	119 (3)
C7A—N3A—N2A	113.0 (3)	C7B—N3B—N2B	112.1 (3)
C5A—C1A—C2A	118.6 (3)	C2B—C1B—C5B	118.9 (3)
C5A—C1A—H1AA	120.7	C2B—C1B—H1BA	120.6
C2A—C1A—H1AA	120.7	C5B—C1B—H1BA	120.6
N1A—C2A—C1A	123.3 (3)	N1B—C2B—C1B	123.6 (4)
N1A—C2A—H2AA	118.3	N1B—C2B—H2BA	118.2
C1A—C2A—H2AA	118.3	C1B—C2B—H2BA	118.2
N1A—C3A—C4A	123.9 (4)	N1B—C3B—C4B	124.0 (4)
N1A—C3A—H3AA	118.1	N1B—C3B—H3BA	118.0
C4A—C3A—H3AA	118.1	C4B—C3B—H3BA	118.0
C3A—C4A—C5A	118.6 (4)	C3B—C4B—C5B	119.0 (4)
C3A—C4A—H4AA	120.7	C3B—C4B—H4BA	120.5
C5A—C4A—H4AA	120.7	C5B—C4B—H4BA	120.5
C1A—C5A—C4A	118.5 (3)	C1B—C5B—C4B	117.7 (3)
C1A—C5A—C6A	123.3 (3)	C1B—C5B—C6B	123.9 (3)
C4A—C5A—C6A	118.1 (3)	C4B—C5B—C6B	118.4 (3)
O1A—C6A—N2A	124.5 (3)	O1B—C6B—N2B	125.0 (3)
O1A—C6A—C5A	121.4 (3)	O1B—C6B—C5B	121.7 (3)
N2A—C6A—C5A	114.2 (3)	N2B—C6B—C5B	113.3 (3)
N3A—C7A—C8A	122.7 (3)	N3B—C7B—C8B	121.3 (4)
N3A—C7A—H7AA	118.7	N3B—C7B—H7BA	119.4
C8A—C7A—H7AA	118.7	C8B—C7B—H7BA	119.4
C9A—C8A—C7A	117.0 (4)	C9B—C8B—C7B	118.8 (4)
C9A—C8A—C12A	123.9 (4)	C9B—C8B—C12B	122.6 (4)
C7A—C8A—C12A	119.1 (3)	C7B—C8B—C12B	118.4 (4)
C8A—C9A—C10A	127.5 (4)	C8B—C9B—C10B	128.5 (5)
C8A—C9A—H9AA	116.3	C8B—C9B—H9BA	115.8
C10A—C9A—H9AA	116.3	C10B—C9B—H9BA	115.8
C9A—C10A—C11A	112.0 (5)	C11B—C10B—C9B	112.3 (6)
C9A—C10A—H10A	109.2	C11B—C10B—H10C	109.1
C11A—C10A—H10A	109.2	C9B—C10B—H10C	109.1
C9A—C10A—H10B	109.2	C11B—C10B—H10D	109.1
C11A—C10A—H10B	109.2	C9B—C10B—H10D	109.1
H10A—C10A—H10B	107.9	H10C—C10B—H10D	107.9
C10A—C11A—H11A	109.5	C10B—C11B—H11D	109.5
C10A—C11A—H11B	109.5	C10B—C11B—H11E	109.5
H11A—C11A—H11B	109.5	H11D—C11B—H11E	109.5
C10A—C11A—H11C	109.5	C10B—C11B—H11F	109.5
H11A—C11A—H11C	109.5	H11D—C11B—H11F	109.5
H11B—C11A—H11C	109.5	H11E—C11B—H11F	109.5
C8A—C12A—H12A	109.5	C8B—C12B—H12D	109.5
C8A—C12A—H12B	109.5	C8B—C12B—H12E	109.5
H12A—C12A—H12B	109.5	H12D—C12B—H12E	109.5
C8A—C12A—H12C	109.5	C8B—C12B—H12F	109.5
H12A—C12A—H12C	109.5	H12D—C12B—H12F	109.5
H12B—C12A—H12C	109.5	H12E—C12B—H12F	109.5
			
C6A—N2A—N3A—C7A	−179.9 (3)	C6B—N2B—N3B—C7B	−175.0 (4)
C3A—N1A—C2A—C1A	−0.3 (6)	C3B—N1B—C2B—C1B	−0.3 (7)
C5A—C1A—C2A—N1A	0.4 (6)	C5B—C1B—C2B—N1B	−0.2 (6)
C2A—N1A—C3A—C4A	0.7 (7)	C2B—N1B—C3B—C4B	1.1 (9)
N1A—C3A—C4A—C5A	−1.3 (8)	N1B—C3B—C4B—C5B	−1.5 (10)
C2A—C1A—C5A—C4A	−0.9 (6)	C2B—C1B—C5B—C4B	−0.2 (6)
C2A—C1A—C5A—C6A	−177.8 (3)	C2B—C1B—C5B—C6B	−177.4 (4)
C3A—C4A—C5A—C1A	1.3 (7)	C3B—C4B—C5B—C1B	0.9 (8)
C3A—C4A—C5A—C6A	178.4 (4)	C3B—C4B—C5B—C6B	178.3 (5)
N3A—N2A—C6A—O1A	−3.1 (6)	N3B—N2B—C6B—O1B	0.6 (6)
N3A—N2A—C6A—C5A	176.2 (3)	N3B—N2B—C6B—C5B	−178.2 (3)
C1A—C5A—C6A—O1A	157.8 (4)	C1B—C5B—C6B—O1B	153.4 (4)
C4A—C5A—C6A—O1A	−19.2 (6)	C4B—C5B—C6B—O1B	−23.8 (6)
C1A—C5A—C6A—N2A	−21.5 (5)	C1B—C5B—C6B—N2B	−27.8 (5)
C4A—C5A—C6A—N2A	161.5 (4)	C4B—C5B—C6B—N2B	155.0 (4)
N2A—N3A—C7A—C8A	−179.5 (4)	N2B—N3B—C7B—C8B	−174.3 (4)
N3A—C7A—C8A—C9A	177.8 (4)	N3B—C7B—C8B—C9B	166.8 (6)
N3A—C7A—C8A—C12A	−2.5 (6)	N3B—C7B—C8B—C12B	−7.8 (7)
C7A—C8A—C9A—C10A	−177.9 (5)	C7B—C8B—C9B—C10B	−176.0 (6)
C12A—C8A—C9A—C10A	2.4 (8)	C12B—C8B—C9B—C10B	−1.7 (11)
C8A—C9A—C10A—C11A	127.7 (6)	C8B—C9B—C10B—C11B	−144.2 (8)

### Hydrogen-bond geometry (Å, °)

**Table:**

*D*—H···*A*	*D*—H	H···*A*	*D*···*A*	*D*—H···*A*
N2B—H1NB···N1A^i^	0.95 (6)	2.08 (6)	2.973 (5)	156 (5)
N2A—H1NA···N1B^ii^	0.83 (6)	2.26 (6)	3.005 (5)	148 (5)
C7B—H7BA···O1A^iii^	0.93	2.51	3.171 (5)	129
C12A—H12B···O1B	0.96	2.48	3.433 (5)	173

Symmetry codes: (i) *x*−1/2, −*y*+3/2, *z*+1/2; (ii) *x*, −*y*+2, *z*−1/2; (iii) *x*−1/2, *y*+1/2, *z*.
